# IS THERE A ROLE FOR BILIODIGESTIVE BYPASS SURGERY IN TREATING CHOLESTASIS IN ADVANCED PANCREATIC CANCER?

**DOI:** 10.1590/0102-6720202400030e1823

**Published:** 2024-09-13

**Authors:** Lucas Cata Preta STOLZEMBURG, Francisco TUSTUMI, Thiago Costa RIBEIRO, Ricardo JUREIDINI, Mauricio Paulin SORBELLO, Fauze MALUF-FILHO, José JUKEMURA, Ulysses RIBEIRO, Guilherme Naccache NAMUR

**Affiliations:** 1 Universidade de São Paulo, Department of Gastroenterology - São Paulo (SP), Brazil.

**Keywords:** Pancreatic Neoplasms, Cholestasis, Biliary Tract Surgical Procedures, Neoplasias Pancreáticas, Colestase, Procedimentos Cirúrgicos do Sistema Biliar

## Abstract

**BACKGROUND:**

The unresectable pancreatic head tumors develop obstructive jaundice and cholestasis during follow-up. Cholestasis is associated with complications and treatment options are endoscopic stenting (ES) and biliary bypass surgery (BBS).

**AIMS:**

The aim of the current study was to compare the safety and efficacy of biliary bypass surgery (BBS) and endoscopic stenting (ES) for cholestasis in advanced pancreas cancer.

**METHODS:**

This is a retrospective cohort of patients with cholestasis and unresectable or metastatic pancreas cancer, treated with BBS or ES. Short and long-term outcomes were evaluated. We considered the need for hospital readmission due to biliary complications as treatment failure.

**RESULTS:**

A total of 93 patients (BBS=43; ES=50) were included in the study. BBS was associated with a higher demand for postoperative intensive care (37 *vs*.10%; p=0.002, p<0.050), longer intensive care unit stay (1.44 standard deviation±2.47 *vs*. 0.66±2.24 days; p=0.004, p<0.050), and longer length of hospital stay (7.95±2.99 *vs*. 4.29±5.50 days; p<0.001, p<0.050). BBS had a higher risk for procedure-related complications (23 *vs*. 8%; p=0.049, p<0.050). There was no difference in overall survival between BBS and ES (p=0.089, p>0.050). ES was independently associated with a higher risk for treatment failure than BBS on multivariate analysis (hazard ratio 3.97; p=0.009, p<0.050).

**CONCLUSIONS:**

BBS is associated with longer efficacy than ES for treating cholestasis in advanced pancreatic cancer. However, the BBS is associated with prolonged intensive care unit and hospital stays and higher demand for intensive care.

## RESUMO

**RACIONAL:**

Os tumores irressecáveis da cabeça do pâncreas desenvolvem icterícia por obstrução e colestase durante o acompanhamento. A colestase está associada a complicações e as opções de tratamento são colocação de prótese endoscópica (PE) e a cirurgia biliodigestiva (CB).

**OBJETIVOS:**

O objetivo do presente estudo é comparar a segurança e eficácia da cirurgia biliodigestiva (CB) com a prótese endoscópica (PE) para colestase em câncer de pâncreas avançado.

**MÉTODOS:**

Este é um estudo coorte retrospectivo de pacientes com colestase e câncer de pâncreas irressecável ou metastático, tratados com CB ou PE. Os resultados de curto e longo prazo foram avaliados. Consideramos a necessidade de readmissão hospitalar por complicações biliares falha no tratamento.

**RESULTADOS:**

Foram incluídos 93 pacientes (CB=43; PE=50). A CB foi associada a maior demanda por cuidados intensivos pós-operatórios (37 *vs*. 10%; p=0,002, p<0.05), maior tempo de permanência na UTI (1,44±2,47 *vs*. 0,66±2,24 dias; p=0,004, p<0.05) e maior tempo de internação hospitalar (7,95±2,99 vs. 4,29±5,5 dias. A CB apresentou maior risco de complicações relacionadas ao procedimento (23 *vs*. 8%; p=0,049, p<0.05). Não houve diferença na sobrevida global entre CB e PE (p=0,089). A PE foi independentemente associada a um maior risco de falha do tratamento do que a CCB na análise multivariada (HR=3,97; p=0,009).

**CONCLUSÕES:**

A CB está associada a uma eficácia mais prolongada do que a PE no tratamento da colestase no câncer pancreático avançado. Entretanto, a CB está associada a internações prolongadas em UTI e hospitalares e maior demanda por cuidados intensivos.

## INTRODUCTION

Pancreatic cancer is associated with poor long-term survival rates. The overall survival rate is still around 7 to 10% in 5 years, and the mean survival rate in unresectable cases is around 11 to 15 months, despite current treatment advancements^5^ ^,^ ^7^ ^,^ ^13^. The unique curative treatment is the R0 resection^5^. However, less than 20% of patients are candidates for curative surgery upon diagnosis, while 29% have locally advanced disease, and around 50% already present metastatic disease^7^. Even among the 20% candidates for surgery, several have non-negligible surgical risk due to poor performance status, significant comorbidities, and advanced age.

Consequently, most patients with pancreatic cancer will require some palliative care to control symptoms, improve quality of life, allow palliative chemotherapy, or prolong survival. Jaundice, chronic pain, and gastroduodenal outflow obstruction are the main symptoms requiring palliation in advanced pancreatic cancer^12^. Up to 80% of unresectable pancreatic head tumors develop obstructive jaundice during follow-up. Cholestasis is associated with complications such as cholangitis, liver and kidney dysfunction, and bleeding^5^ ^,^ ^12^. The main treatment options for treating cholestasis are endoscopic stenting (ES), percutaneous transhepatic drainage, and biliary bypass surgery (BBS) as well as choledochal-jejunal and hepatic-jejunal biliodigestive diversion^5^ ^,^ ^8^ ^,^ ^12^.

The decision on the biliary drainage strategy should be based on the patient’s and tumor’s individual characteristics, costs, and availability of resources^5^ ^,^ ^9^. The goal is to provide symptom relief with a low risk for procedure-related complications while providing long-term efficacy. The treatment efficacy can be measured by the time free from biliary reinterventions, redo procedures, biliary complications, and symptom recurrence, which is closely related to the long-term patency of the biliary tree. With the improvement of endoscopic stents and ES expertise, the call for the adoption of less invasive measures has been increasing in the last few years^8^.

The aim of the current study was to compare the safety and efficacy of BBS and ES for advanced pancreatic cancer.

## METHODS

### Study design

This is a single-center retrospective cohort of patients with advanced pancreatic cancer treated between 2009 and 2016.

### Ethics approval

The local institutional Ethic Committee approved the study and waived consent to participate (number: #0084/0001).

### Eligibility

Patients with cholestasis and diagnosis of unresectable (locally advanced tumor) or metastatic (stage IV) ductal adenocarcinoma of the pancreas and treated by BBS or ES were included.

Patients with other periampullary neoplasms and those in whom it was not possible to perform derivative surgery or the insertion of endoscopic stenting were excluded.

### Preoperative evaluation

Before the procedures, patients were evaluated with imaging tests (abdominal tomography, magnetic resonance imaging, and/or endoscopic ultrasound), serum laboratory tests (blood count, tumor markers, basic biochemistry, total bilirubin and fractions, albumin), and preoperative clinical and anesthetic assessments.

### Procedures

After a detailed assessment, the biliary intervention was defined individually. Some pretreatment variables were pondered for the decision regarding cholestasis treatment. Patients with loss of performance status - ECOG (Eastern Cooperative Oncology Group) 3 or 4 - or with cholangitis usually underwent ES. Conversely, a BBS was usually performed in patients with duodenal obstruction or if unresectability was determined during the operation. Any decision was shared with patient and family.

Surgical drainage consisted of performing a hepatic-jejunal or common-jejunal biliodigestive diversion, with continuous end-to-side anastomosis using 5-0 or 6-0 monofilament polydioxanone suture (PDS), with the “parachute” technique, and a biliodigestive loop of approximately 50 cm, transmesocolic, and laterolateral enteroenteroanastomosis performed with a 75 mm linear stapler. Two experienced hepatobiliary surgeons performed the procedures.

Endoscopic drainage was performed using self-expandable metal stenting inserted by retrograde endoscopic cholangiography. The procedure was performed with a duodenoscope, with visualization of the duodenal papilla and selective catheterization of the bile ducts with a Teflon guide wire, followed by cholangiography with iodinated contrast medium. Usually, after endoscopic papillotomy, the stent is inserted with control radioscopy at the end of the procedure.

### Follow-up

All patients were followed up until death.

### Data extraction

Data were collected from electronic medical records. The patient’s pretreatment baseline characteristics were extracted, including age, sex, body mass index (BMI), American Society of Anesthesiologists physical status (ASA score), and ECOG. In addition, laboratory serum tests and staging imaging tests were assessed.

### Outcomes

The following outcomes were extracted:

- In-hospital death;
- Length of hospital stay;
- Length of Intensive Care Unit (ICU) stay;
- Overall procedure-related complications;
- Procedure-related bleeding;
- Post-procedure palliative chemotherapy;
- Overall survival; and
- Treatment failure.

We considered treatment failure as the need for hospital readmission due to biliary complications, such as cholestasis and cholangitis.

### Statistical analysis

STATA 16.1 software (StataCorp LLC, 4905 Lakeway Dr, College Station, TX 77845, United States) was used to conduct the analyses.

Qualitative characteristics were investigated by applying Fisher’s exact tests. Quantitative characteristics were described according to groups using mean and standard deviation (SD) and assessed with Mann-Whitney tests. Survival curves were compared with the log-rank test and presented by Kaplan-Meier curves. The Cox regression model was used for time-to-event analysis. Associations with p<0.100 in univariate analysis were selected for multivariate analysis. Tests were carried out with a significance level of 5%.

As a sensitivity analysis, we performed a propensity score matching (PSM) analysis 1:1, matching the covariables that most likely influenced the outcomes, including age, level of serum direct bilirubin, level of carbohydrate antigen 19-9 (CA 19-9), serum albumin, and pretreatment ECOG.

## RESULTS

In total, 93 patients were included in the study. Among them, 43 underwent BBS and 50 ES. The baseline characteristics of each group are presented in Table 1. ES group had patients with higher levels of direct bilirubin (p=0.040) and worse ECOG (p=0.019), while BBS had more frequent gastroduodenal outflow obstruction (26 *vs.* 6%; p=0.017). There was no significant difference in age, sex, BMI, albumin, CA 19-9, or history of previous biliary drainage (p>0.050). Besides, neither group had any significant difference in distant metastasis or locally advanced tumors (p>0.050).

**Table 1 -:** Baseline characteristics of the included patients. Results were presented before and after propensity score matching analysis. A p-value<0.050 was considered significant.

		Before PSM			After PSM		
		Procedure			Procedure		
		BBS (n=43)	ES (n=50)	p-value	BBS (n=26)	ES (n=26)	p-value
Age	(>65 years old)	22 (43)	28 (50)	0.641	14 (26)	13 (26)	0.781
Sex	(Female)	26 (43)	26 (50)	0.412	18 (26)	14 (26)	0.254
BMI	(<20 kg/m^2^)	11 (43)	10 (47)	0.630	7 (26)	3 (26)	0.159
Direct bilirubin	(>10 mg/dL)	15 (42)	27 (47)	0.040	11 (26)	11 (26)	>0.999
Serum albumin	(<3.5 g/dL)	17 (36)	28 (42)	0.083	14 (26)	17 (26)	0.397
CA 19-9 (U/mL)	(>1000 U/mL)	13 (37)	17 (45)	0.805	6 (22)	7 (23)	0.815
Gastroduodenal outflow obstruction		11 (43)	3 (50)	0.008	8 (26)	0 (26)	0.002
Locally advanced tumor		34 (43)	37 (50)	0.566	20 (26)	21 (26)	0.734
Distant metastasis (stage IV)		21 (43)	26 (50)	0.761	14 (26)	11 (26)	0.405
Previous biliary drainage		23 (43)	31 (50)	0.407	12 (26)	18 (26)	0.092
ASA score	(III/IV)	15 (43)	21 (49)	0.434	9 (26)	8 (26)	0.768
ECOG	(3/4)	2(36)	13 (49)	0.012	2 (26)	2 (26)	>0.999

PSM: propensity score matching; BBS: biliary bypass surgery; ES: endoscopic stenting; BMI: body mass index; CA: carcinoembryonic antigen; ASA: American Society of Anesthesiologists; ECOG: Eastern Cooperative Oncology Group.

**Figure 1 -:**
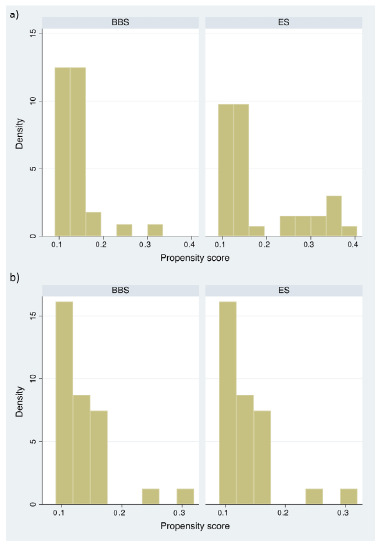
Histogram showing the distribution of the propensity scores before (a) and after (b) 1:1 matching for the covariables age, direct bilirubin, albumin levels, and Eastern Cooperative Oncology Group.

BBS: biliary bypass surgery; ES: endoscopic stenting.

After PSM analysis, the main covariables potentially associated with the outcomes were balanced between groups, except for gastroduodenal outflow obstruction (Figure 1).

### Outcomes

BBS was associated with a higher risk for complications (23 *vs.* 8%; p=0.049, p<0.050), higher demand for postoperative intensive care (37 *vs.* 10%; p=0.002, p<0.050), longer ICU stay (1.44±2.47 *vs.* 0.66±2.24 days; p=0.004, p<0.050), and longer length of hospital stay (7.95±2.99 *vs.* 4.29±5.5 days; p<0.001, p<0.050). There was no significant difference in bleeding and in-hospital death (p>0.050). In the BBS group, patients were more likely to undergo palliative chemotherapy after surgery (74 *vs.* 52%; p=0.033, p<0.050).

After matching, demand for ICU, ICU stay, and length of hospital stay were significantly higher in the BBS group, while procedure-related complications and palliative chemotherapy were not (Table 2).

**Table 2 -:** Comparison between the outcomes of the procedures bypass biliary surgery and endoscopic stenting for cholestasis in pancreatic cancer patients. Results were presented before and after propensity score matching analysis. A p-value<0.050 was considered significant.

	Before PSM			After PSM		
	Procedure			Procedure		
	BBS (n=43)	ES (n=50)	p-value	BBS (n=26)	ES (n=26)	p-value
ICU	16 (43)	5 (48)	0.002	11 (26)	3 (26)	0.042
Length of ICU stay (days)	1.44±2.47 (43)	0.66±2.24 (50)	0.004	1.9±2.9 (26)	0.8±2.2 (26)	0.024
Length of hospital stay (days)	7.95±2.99 (40)	4.29±5.5 (49)	<0.001	8.2±3.3 (24)	3.3±3.8 (26)	<0.001
Overall procedure-related complications	10 (43)	4 (48)	0.049	0 (26)	0 (26)	>0.999
Bleeding	2 (43)	2 (50)	0.877	1 (26)	1 (26)	>0.999
In-hospital death	5 (43)	6 (50)	0.956	3 (26)	0 (26)	0.074
Palliative chemotherapy	32 (43)	26 (50)	0.026	19 (26)	17 (26)	0.548

PSM: propensity score matching analysis; BBS: bypass biliary surgery; ES: endoscopic stenting; ICU: intensive care unit.

### Overall survival

The mean survival in the studied cohort was 11.6 months (±10.2). There was no difference in overall survival between BBS and ES (log-rank p=0.089, p<0.050). On Cox regression, direct bilirubin (hazard ratio [HR] 1.53; p<0.049, p<0.050) and serum albumin (HR 1.53; p=0.035, p<0.050) levels, ECOG (HR 2.99; p<0.001), ASA score (HR 1.64; p=0.023, p<0.050), and the presence of distant metastasis (HR 1.76; p=0.010) were associated with survival in univariate analysis. On multivariate analysis, gastroduodenal obstruction (HR 2.77; p=0.010, p>0.050), distant metastasis (HR 2.39; p=0.003, p<0.050), and ASA score (HR 2.35; p=0.006, p<0.050) kept significant.

**Figure 2 -:**
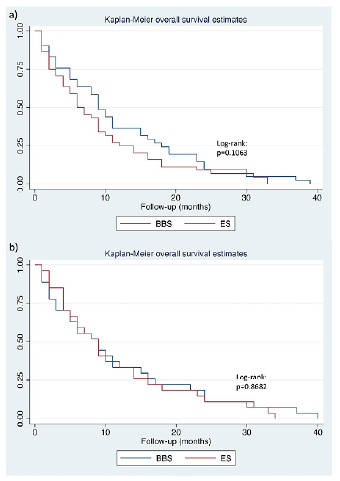
Overall survival before (a) and after (b) propensity score matching.

BBS: biliary bypass surgery; ES: endoscopic stenting.

In the PSM analysis, only direct bilirubin (HR 1.84; p=0.037, p<0.050) was associated with survival in univariate analysis. In multivariate analysis, serum albumin (HR 1.79; p=0.047, p<0.050) was considered an independent prognostic variable (Figure 2 and Table 3).

**Table 3 -:** Overall survival for treatment of advanced pancreatic cancer with biliary bypass surgery and endoscopic stenting before and after propensity score matching. Associations with p-value<0.100 in univariate analysis were selected for multivariate analysis. A p-value<0.050 was considered significant.

Overall survival									
Before PSM		Univariate				Multivariate			
		95%CI				95%CI			
		HR	LL	UL	p-value	HR	LL	UL	p-value
Age	(>65 years old)	1.23	0.81	1.86	0.336				
Sex	(Female *vs.* Male)	1.03	0.68	1.57	0.876				
BMI	(<20 kg/m^2^)	1.29	0.79	2.12	0.308				
Direct bilirubin	(>10 mg/dL)	1.53	1.01	2.34	0.049	1.29	0.65	2.57	0.461
Serum albumin	(<3.5 g/dL)	1.64	1.03	2.60	0.035	1.35	0.77	2.38	0.292
CA 19-9	(>1000 U/mL)	1.01	0.63	1.59	0.986				
Gastroduodenal outflow obstruction		1.70	0.96	3.02	0.071	2.77	1.28	5.99	0.010
Locally advanced tumor		1.22	0.75	2.00	0.420				
Distant metastasis (stage IV)		1.76	1.15	2.70	0.010	2.39	1.35	4.20	0.003
Previous biliary drainage		0.69	0.45	1.05	0.084	0.6	0.31	1.19	0.143
ASA score	(III/IV vs. I/II)	1.64	1.07	2.54	0.023	2.35	1.28	4.31	0.006
ECOG	(3/4 *vs*. 0/1/2)	2.99	1.68	5.33	<0.001	2.15	0.99	4.70	0.055
Procedure	(ES *vs.* BBS)	1.43	0.94	2.17	0.092	1.75	0.98	3.16	0.059
**After PSM**		**Univariate**				**Multivariate**			
		**95%CI**				**95%CI**			
		**HR**	**LL**	**UL**	**p-value**	**HR**	**LL**	**UL**	**p-value**
Age	(>65 years old)	1.17	0.67	2.05	0.581				
Sex	(Female vs. Male)	1.06	0.60	1.88	0.831				
BMI	(<20 kg/m^2^)	1.51	0.75	3.03	0.250				
Direct bilirubin	(>10 mg/dL)	1.84	1.04	3.25	0.037	1.60	0.68	3.77	0.284
Serum albumin	(<3.5 g/dL)	1.73	0.98	3.05	0.061	1.79	1.01	3.17	0.047
CA 19-9	(>1000 U/mL)	0.90	0.46	1.76	0.759				
Gastroduodenal outflow obstruction		1.79	0.84	3.86	0.132				
Locally advanced tumor		1.77	0.88	3.58	0.111				
Distant metastasis (stage IV)		1.75	0.98	3.13	0.056	1.67	0.93	3.00	0.089
Previous biliary drainage		0.62	0.35	1.09	0.098	0.91	0.39	2.12	0.836
ASA score	(III/IV *vs.* I/II)	1.38	0.76	2.52	0.295				
ECOG	(3/4 *vs.* 0/1/2)	1.62	0.58	4.55	0.357				
Procedure	(ES *vs.* BBS)	1.24	0.71	2.18	0.445				

PSM: propensity score matching; 95%CI: 95% confidence interval; HR: hazard ratio; LL: lower limit; UL: upper limit; BMI: body mass index; CA: carcinoembryonic antigen; ASA: American Society of Anesthesiologists; ECOG: Eastern Cooperative Oncology Group; BBS: biliary bypass surgery; ES: endoscopic stenting.

### Treatment failure

**Figure 3 -:**
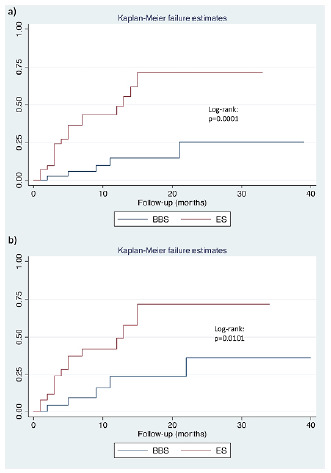
Treatment failure for biliary bypass surgery (BBS) and endoscopic stenting (ES) before (a) and after (b) propensity score matching. We considered treatment failure as the need for hospital readmission due to biliary complications such as cholestasis, cholangitis, and treatment of biliary complications.

BBS was significantly associated with a lower risk for readmission for biliary complications during follow-up than ES (log-rank p<0.001). In regression univariate analysis, the procedure was associated with the risk for treatment failure (ES *vs.* BBS: HR 6.09; p<0.001). This association was also significant in multivariate analysis (HR 3.97; p=0.009, p<0.050). The analysis of the matched groups showed that procedure and pretreatment direct bilirubin levels were significantly associated with the risk of treatment failure (Figure 3 and Table 4).

**Table 4 -:** Treatment failure for biliary bypass surgery and endoscopic stenting before and after propensity score matching. We considered treatment failure as the need for hospital readmission due to biliary complications such as cholestasis, cholangitis, and treatment of biliary complications. Associations with p-value<0.100 in univariate analysis were selected for multivariate analysis. A p-value<0.050 was considered significant.

Before PSM		Univariate				Multivariate			
		95%CI				95%CI			
		HR	LL	UL	p-value	HR	LL	UL	p-value
Age	(>65 years old)	1.21	0.56	2.66	0.627				
Sex	(Female *vs.* Male)	0.87	0.4	1.89	0.719				
BMI	(<20 kg/m^2^)	1.21	0.48	3.07	0.677				
Direct bilirubin (mg/dL)	(>10 mg/dL)	1.79	0.83	3.88	0.139				
Serum albumin	(<3.5 g/dL)	2.39	0.97	5.89	0.059	1.72	0.68	4.36	0.251
CA 19-9 (U/mL)	(>1000 U/mL)	1.26	0.55	2.91	0.581				
Gastroduodenal outflow obstruction		0.35	0.05	2.59	0.303				
Locally advanced tumor		1.36	0.51	3.62	0.534				
Distant metastasis (stage IV)		1.73	0.79	3.76	0.167				
Previous biliary drainage		0.55	0.25	1.18	0.126				
ASA score	(III/IV *vs.* I/II)	1.11	0.48	2.60	0.804				
ECOG	(3/4 *vs.* 0/1/2)	1.69	0.50	5.73	0.402				
Procedure	(ES *vs.* BBS)	6.09	2.26	16.36	<0.001	3.97	1.40	11.12	0.009
Treatment failure									
**After PSM**		**Univariate**				**Multivariate**			
		**95%CI**				**95%CI**			
		**HR**	**LL**	**UL**	**p-value**	**HR**	**LL**	**UL**	**p-value**
Age	(>65 years old)	0.85	0.33	2.16	0.727				
Sex	(Female *vs.* Male)	0.98	0.38	2.53	0.961				
BMI	(<20 kg/m^2^)	1.32	0.43	4.05	0.624				
Direct bilirubin	(>10 mg/dL)	3.31	1.27	8.60	0.014	4.25	1.56	11.62	0.005
Serum albumin	(<3.5 g/dL)	1.45	0.56	3.75	0.446				
CA 19-9	(>1000 U/mL)	1.87	0.67	5.19	0.232				
Gastroduodenal outflow obstruction		0.01	0	0.01	>0.999				
Locally advanced tumor		1.28	0.42	3.92	0.660				
Distant metastasis (stage IV)		2.03	0.80	5.28	0.138				
Previous biliary drainage		0.55	0.21	1.40	0.208				
ASA score	(III/IV *vs.* I/II)	0.98	0.35	2.76	0.968				
ECOG	(3/4 *vs.* 0/1/2)	1.86	0.42	8.14	0.410				
Procedure	(ES *vs.* BBS)	7.72	2.14	27.93	0.002	9.41	2.50	35.46	0.001

PSM: propensity score matching; 95%CI: 95% confidence interval; HR: hazard ratio; LL: lower limit; UL: upper limit; BMI: body mass index; CA: carcinoembryonic antigen; ASA: American Society of Anesthesiologists; ECOG: Eastern Cooperative Oncology Group; BBS: biliary bypass surgery; ES: endoscopic stenting.

## DISCUSSION

The results of the present study showed that BBS is more effective in treating cholestasis in advanced pancreatic cancer but demands a higher usage of intensive care resources and longer hospital stays. However, the decision between ES and BBS should be individualized based on the patient’s overall health, the extent of the disease, and the goals of care. ES is often preferred for its minimally invasive nature, especially in patients with limited life expectancy or significant comorbidities. On the other hand, BBS may be considered in cases where a more durable solution is necessary or when ES is not technically feasible^4^.

Both ES and BBS carry their own set of risks and potential complications. Patients should be informed about these risks so they can make well-informed decisions in collaboration with their healthcare providers. The patient’s preferences and values should be considered, and discussions should include a clear understanding of the potential benefits and risks associated with each treatment option. Ultimately, a multidisciplinary approach involving gastroenterologists, endoscopists, oncologists, and surgeons is crucial for making informed decisions regarding managing cholestasis in advanced pancreatic cancer^4^. Regular follow-up and ongoing communication among the healthcare team are essential to monitor treatment efficacy and address any potential complications^6^.

In our research, we observed that BBS was associated with a higher risk of complications compared to ES. This heightened risk is likely attributable to the compromised baseline health and performance status of the patients, mainly in the ES group. Upon conducting matching analyses, BBS was linked to complication risks as low as those seen with ES. Consequently, selecting patients for the appropriate treatment strategy is the key to mitigating procedure-related risks. A meta-analysis comprising five studies found that ES was associated with a lower risk of complications. However, the included studies had significant clinical and statistical heterogeneity^1^. A study protocol for a Cochrane systematic review comparing BBS and ES was published^8^, although the final results were not presented. Future publications from this protocol should provide updates on complications and risk differences.

After matching, gastroduodenal flow obstruction was found to differ between the groups. This difference is because malignant gastroduodenal outflow obstruction typically demands a surgical approach. Obstruction may pose challenges in accessing the duodenal papilla by endoscopy; therefore, surgery can possibly solve biliary and gastric obstruction. Considering it is well established that gastroduodenal flow obstruction is associated with protein-calorie malnutrition, we performed a sensitivity analysis matching albumin serum level to reduce the potential heterogeneity between studied groups^15^ ^,^ ^18^. The placement of an endoscopic duodenal stent followed by a transpapillary endoscopic biliary stent could be an alternative to the biliary bypass in gastroduodenal flow obstruction^11^. When the duodenal stent overlaps the papilla, the biliary tree can be accessed by the rendezvous technique, either by guidance with percutaneous transhepatic biliary drainage or endoscopic ultrasound. Rizzo et al. performed a systematic review of endoscopic strategies for concomitant malignant biliary obstruction and gastric outlet obstruction. The authors highlighted the outstanding role of endoscopic ultrasound-guided strategies and reported that endoscopic ultrasound-guided biliary stenting was technically successful in 96% of patients and clinically successful in 85%^14^. Only future controlled trials can provide the best evidence to support either BBS or endoscopic duodenal and biliary stenting for simultaneous biliary and duodenal obstruction.

Assessing the effectiveness of any biliary intervention requires careful consideration of not only short-term outcomes but also long-term benefits and risks. The challenge is to capture a comprehensive picture of the impact of each intervention on patients’ overall well-being over time. Consequently, the ideal biliary intervention is the one that provides improved quality of life for long periods, mitigating symptoms. In this sense, a metal stent seems to be a better option than a plastic stent for patients with advanced non-curable pancreatic cancer due to the long efficacy of metal stents. In a randomized controlled trial, Walter et al. compared metal *vs.* plastic stents for malignant biliary obstruction. The authors concluded that self-expandable metal stents result in better scores for general and disease-specific health-related quality of life over time^19^. The lower quality of life in the plastic stent group is attributed to its inferior durability, leading to a higher frequency of dysfunction and, consequently, higher rates of symptom recurrence over time.

Determining appropriate measures to evaluate biliary drainage efficacy is challenging. Outcomes can include factors such as survival, quality of life, and relief of cholestasis symptoms. Defining standardized and clinically meaningful criteria for these outcomes is crucial to a study’s meaningfulness. For this point, we considered treatment success as the time free of hospitalization due to biliary complications in a longitudinal assessment. In this sense, our study showed that BBS had better efficacy than ES over time. Bliss et al. chose reintervention as the primary outcome in their matched cohort. The authors found that ES was associated with a significantly higher risk for reintervention than BBS (20.3 *vs.* 4.5%)^3^. However, the authors acknowledged their study’s limitations since they included both plastic and metal stents in their analyses. Besides, their study had limited follow-up data, impacting longitudinal inferences. Scott et al., in an observational study with 56 advanced pancreatic cancer patients, found that ES was associated with a higher risk for readmission than BBS (39.4 *vs.* 13%) and lower overall survival (135 *vs.* 382 days)^16^. The lack of multiple regression analysis or matching groups prone the study to the risk of selection bias since patients with poor clinical status are usually addressed to endoscopic palliation.

Our initial analysis suggested that ES was more effective at enabling palliative chemotherapy, which could potentially impact long-term survival. Successfully undergoing chemotherapy is a critical factor in the management of advanced pancreatic neoplasms. Chemotherapy addresses systemic disease and enhances overall survival^17^. The decision to administer chemotherapy to patients with cholestasis is personalized and necessitates a thorough assessment of the associated risks and benefits. Typically, cholestasis hinders the use of palliative chemotherapy^2^. The liver plays a vital role in metabolizing and eliminating numerous chemotherapy drugs, and cholestasis may affect the clearance and metabolism of these drugs^10^. In consequence, theoretically, the effectiveness of biliary drainage could enhance palliative chemotherapy’s tolerability. However, upon conducting sensitivity analysis and matching for performance status (ECOG), we observed that the rate of palliative chemotherapy was similar in both groups. Besides, the discretion between BBS and ES did not interfere with overall survival.

This retrospective study has some inherent limitations to consider when interpreting its findings. Retrospective studies are prone to selection bias, as treatment choice is often influenced by factors such as patient characteristics, physician preferences, and institutional practices. Patients with advanced pancreatic cancer are a heterogeneous group in terms of disease stage, comorbidities, and overall health. Patients may be selected for a particular treatment based on their overall health status, comorbidities, and the extent of the disease. In general, patients in worse clinical conditions underwent endoscopic treatment since surgical risk would be higher for them. This can introduce a bias that affects the generalizability of the study results. We implemented a comprehensive analytical approach to mitigate the risk of bias and enhance the robustness of our findings. Firstly, multivariate analysis was conducted to account for the influence of various confounding factors on treatment outcomes, providing a more nuanced understanding of the results. Additionally, a sensitivity analysis was employed to assess the stability and consistency of our findings by matching baseline variables that could potentially interfere with the main investigated outcomes. Potential confounding covariables cannot be all addressed, and only future randomized trials will provide the highest grade of evidence.

## CONCLUSIONS

BBS is associated with longer efficacy than ES for treating cholestasis in advanced pancreatic cancer. However, the surgical approach is associated with prolonged ICU and hospital stays and higher demand for intensive care. The choice between BBS or ES does not influence long-term survival.
